# Supplementary material for: IL-21/23 axis modulates inflammatory cytokines and RANKL expression in RA CD4+ T cells via p-Akt1 signaling
Source: Front Immunol. 2023 Sep 21;14:1235514. doi: 10.3389/fimmu.2023.1235514 (PMC10551441; doi:10.3389/fimmu.2023.1235514)
Supplement: Supplementary file 1 [file DataSheet_1.pdf]

**Reagent**	**Catalogue No.**
Milliplex MAP Human Th17 Magnetic Bead Panel-Immunology Multiplex Assay	Millipore HTH17MAG-14K
Dynabeads ™ Untouched Human CD4 T cells Kit	Invitrogen 11346D
BD Cytofix/Cytoperm™	BD Biosciences 554714
eBiosciences™ FoxP3/Transcription Factor Staining Buffer Set	Invitrogen 00-5523-00
Poly-L-Lysine 0.01	Sigma Aldrich P4707
ProLong™ Gold Antifade Mountant with DAPI	Invitrogen P36935
Hoechst Stain	Sigma Aldrich H6024
Hyaluronidase from bovine testes	Sigma Aldrich H3506-100MG
RPMI 1640	PAN-BIOTECH P04-16520
Fetal Bovine Serum	PAN-BIOTECH P30-1402
Zombie Violet™ Fixable Viability Kit	Biolegend 423113
DPBS, w/0: Ca and Mg	PAN BIOTECH P04-36500
Akt1/2 Kinase Inhibitor	Sigma Aldrich A6730-5MG
FlowJO Version 10.8	BD Biosciences
Graph Pad Prism 9	Dotmatics Pvt Ltd.

**Recombinant Human Cytokines**	**Catalogue No.**
IL-6 Protein Human Recombinant	Prospec Bio<br>CYT-213
IL-1β Protein Human Recombinant	Prospec Bio<br>CYT-208
TGF-β Protein Human Recombinant	Prospec Bio<br>CYT-716
IL-21 Protein Human Recombinant	Prospec Bio<br>CYT-408
IL-23 Protein Human Recombinant	Prospec Bio<br>CYT-050

**Fluorophore-tagged antibody**	**Catalogue No. Antibody**	**Isotype**
IFN-γ BV480	BD Biosciences 566100	BD Biosciences 565652
TNF-α PECF594	BD Biosciences 562784	BD Biosciences 562292
IL-21R PECF594	BD Biosciences 564122	BD Biosciences 562292
RANKL APC	Biolegend 347508	Biolegend 400322
IL-23R PE	R&D FAB14001P	R&D IC0041P
CD3 AF700	Biolegend 317340	Biolegend 400248
IL-17 PerCPCy5.5	BD Biosciences 560799	BD Biosciences 552834
IL-23p40 PE	Biolegend 501806	R&D IC002P
RANKL PE	Biolegend 347503	Biolegend 400311
IL-23p19 PE	R&D IC17161P	R&D IC0041P
IL-21 AF647	BD Biosciences 562043	BD Biosciences 557714
GMCSF AF647	BD Biosciences 562257	BD Biosciences 557906
T-bet AF488	BD Biosciences 561266	Ebioscience 53-4714-42
RorγT BV650	BD Biosciences 563424	BD Biosciences 563437
IL-10 PE	BD Biosciences 559330	BD Biosciences 559317
CD4 PECy7	Biolegend 300512	Ebioscience 25-4714-42
pAkt1 APC	Ebioscience 17-9715-42	Ebioscience 17-4724-81
