# Supplementary material for: IL-21/23 axis modulates inflammatory cytokines and RANKL expression in RA CD4+ T cells via p-Akt1 signaling
Source: Front Immunol. 2023 Sep 21;14:1235514. doi: 10.3389/fimmu.2023.1235514 (PMC10551441; doi:10.3389/fimmu.2023.1235514)
Supplement: Supplementary file 2 [file Presentation_1.pptx]

## Slide 1
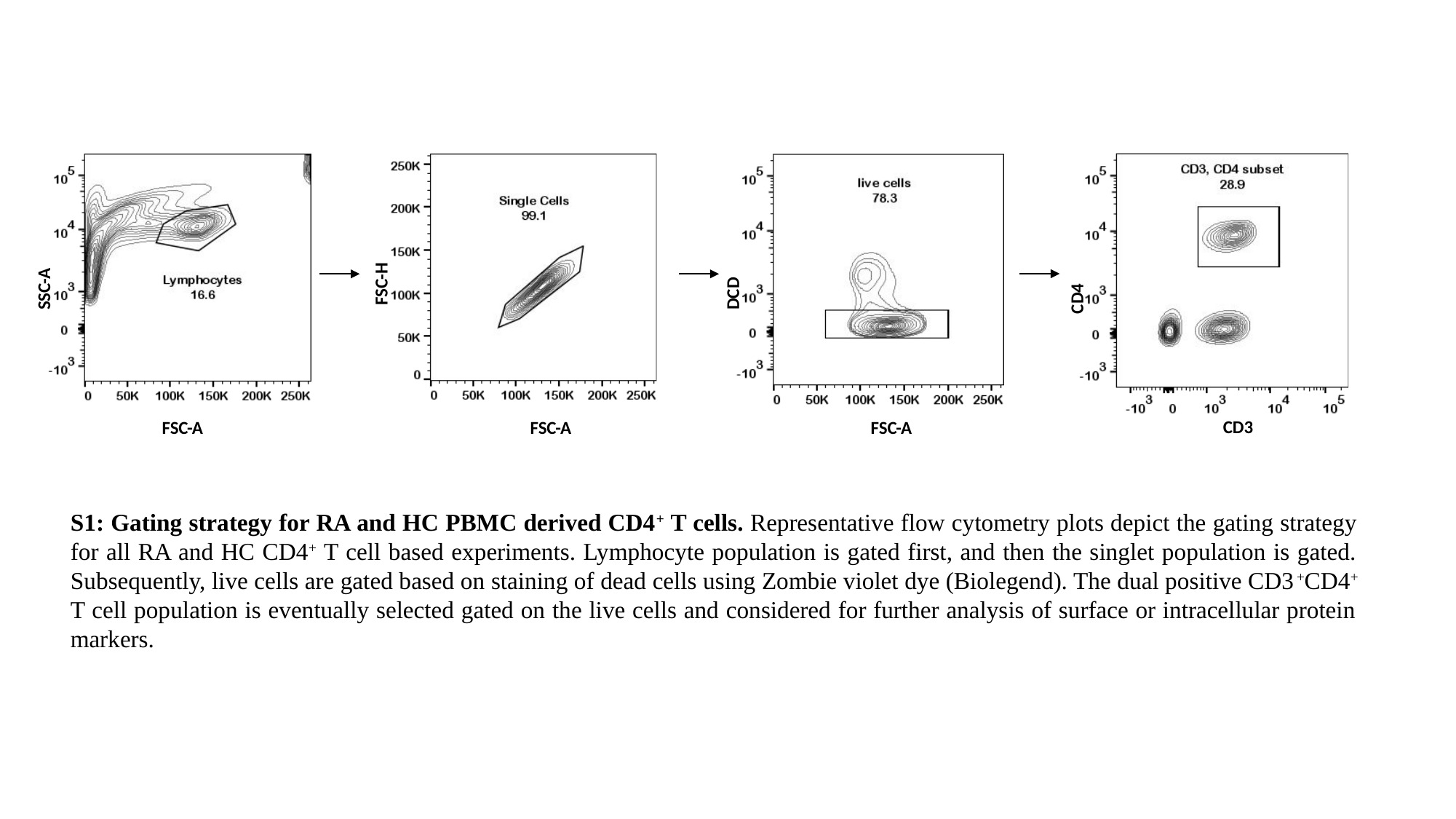

FSC-H
SSC-A
DCD
CD4
CD3
FSC-A
FSC-A
FSC-A
S1: Gating strategy for RA and HC PBMC derived CD4+ T cells. Representative flow cytometry plots depict the gating strategy for all RA and HC CD4+ T cell based experiments. Lymphocyte population is gated first, and then the singlet population is gated. Subsequently, live cells are gated based on staining of dead cells using Zombie violet dye (Biolegend). The dual positive CD3+CD4+ T cell population is eventually selected gated on the live cells and considered for further analysis of surface or intracellular protein markers.

## Slide 2
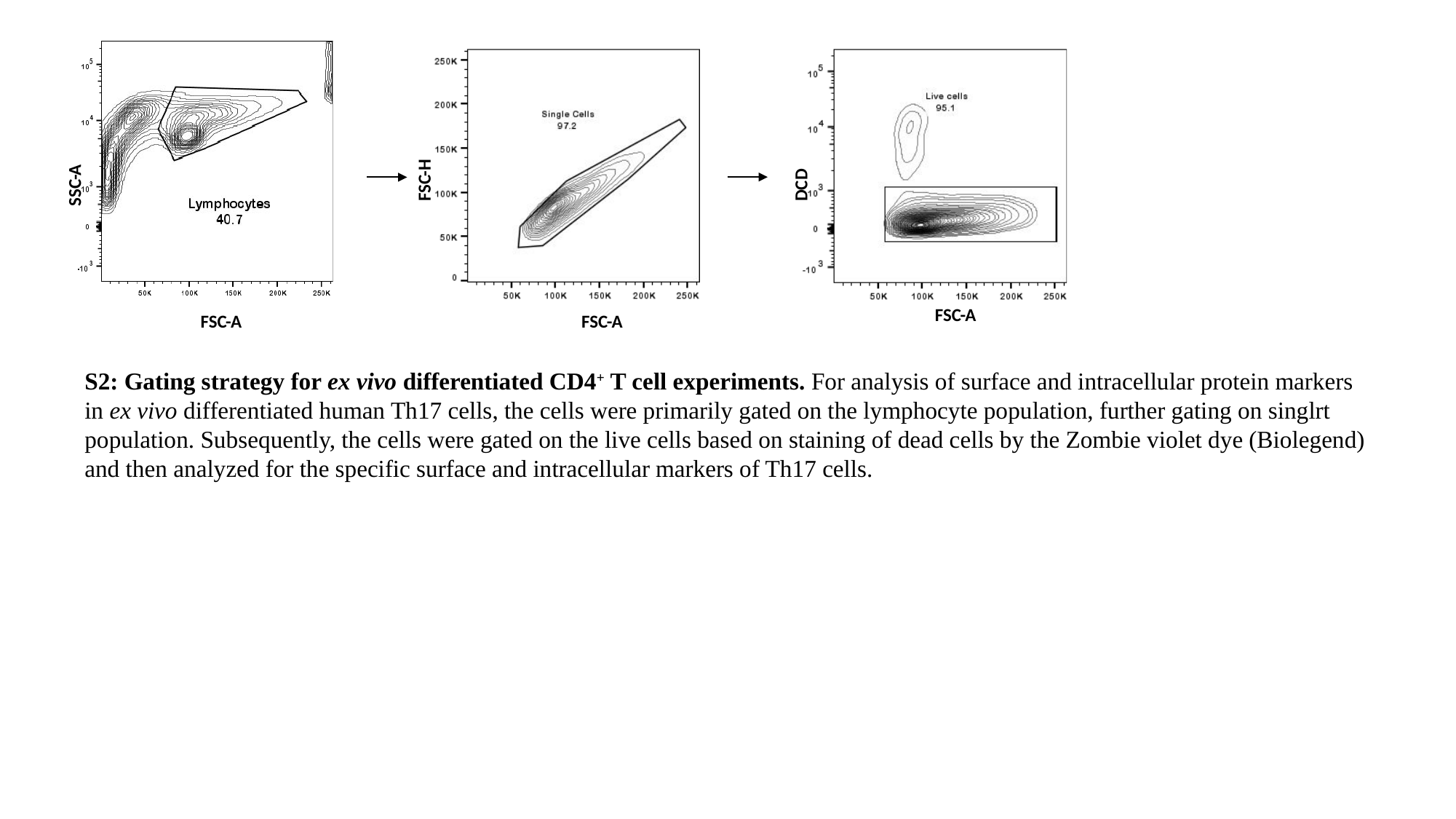

DCD
FSC-H
SSC-A
FSC-A
FSC-A
FSC-A
S2: Gating strategy for ex vivo differentiated CD4+ T cell experiments. For analysis of surface and intracellular protein markers in ex vivo differentiated human Th17 cells, the cells were primarily gated on the lymphocyte population, further gating on singlrt population. Subsequently, the cells were gated on the live cells based on staining of dead cells by the Zombie violet dye (Biolegend) and then analyzed for the specific surface and intracellular markers of Th17 cells.

## Slide 3
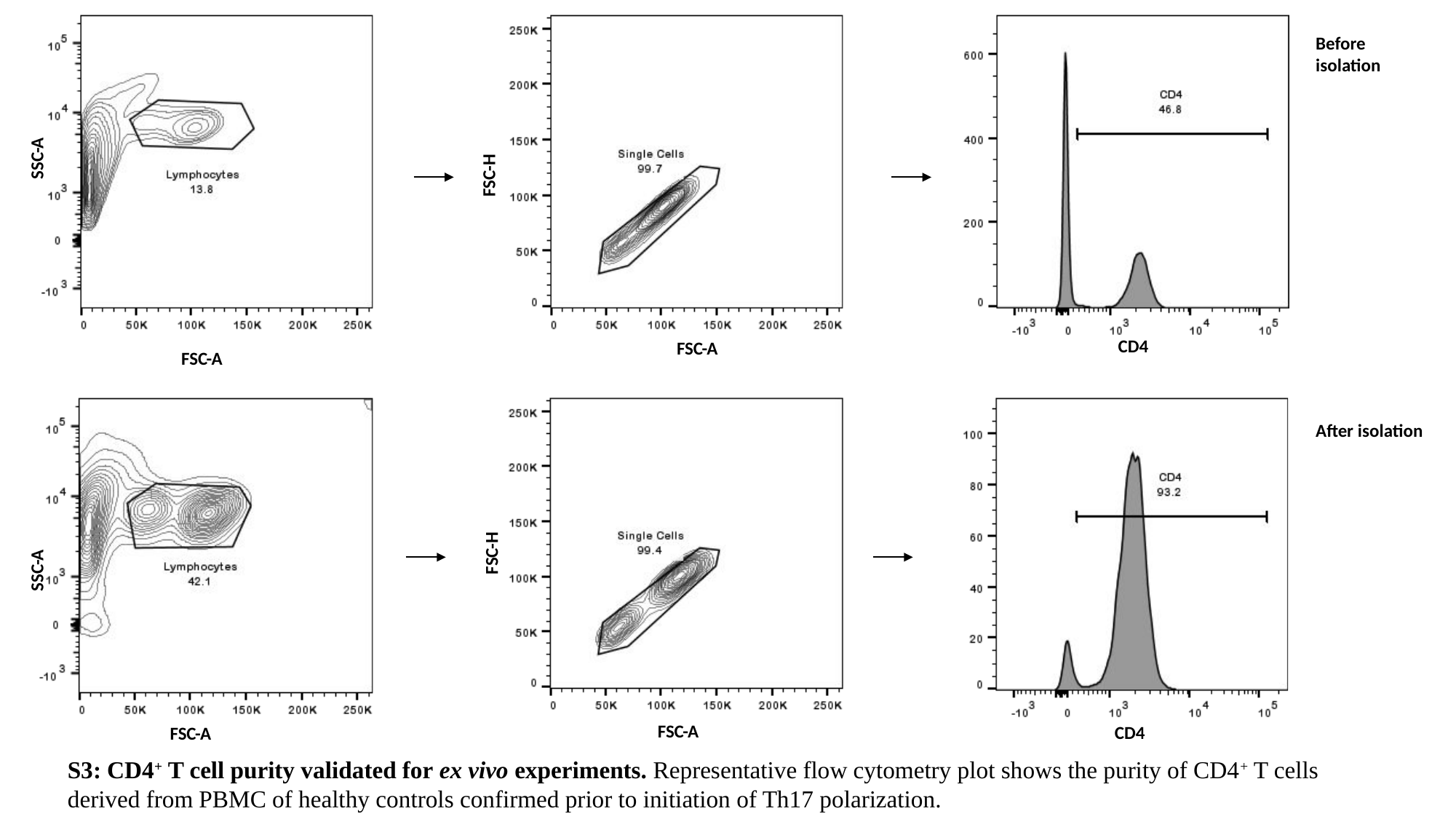

SSC-A
FSC-H
CD4
FSC-A
FSC-A
Before isolation
FSC-H
SSC-A
FSC-A
CD4
FSC-A
After isolation
S3: CD4+ T cell purity validated for ex vivo experiments. Representative flow cytometry plot shows the purity of CD4+ T cells derived from PBMC of healthy controls confirmed prior to initiation of Th17 polarization.

## Slide 4
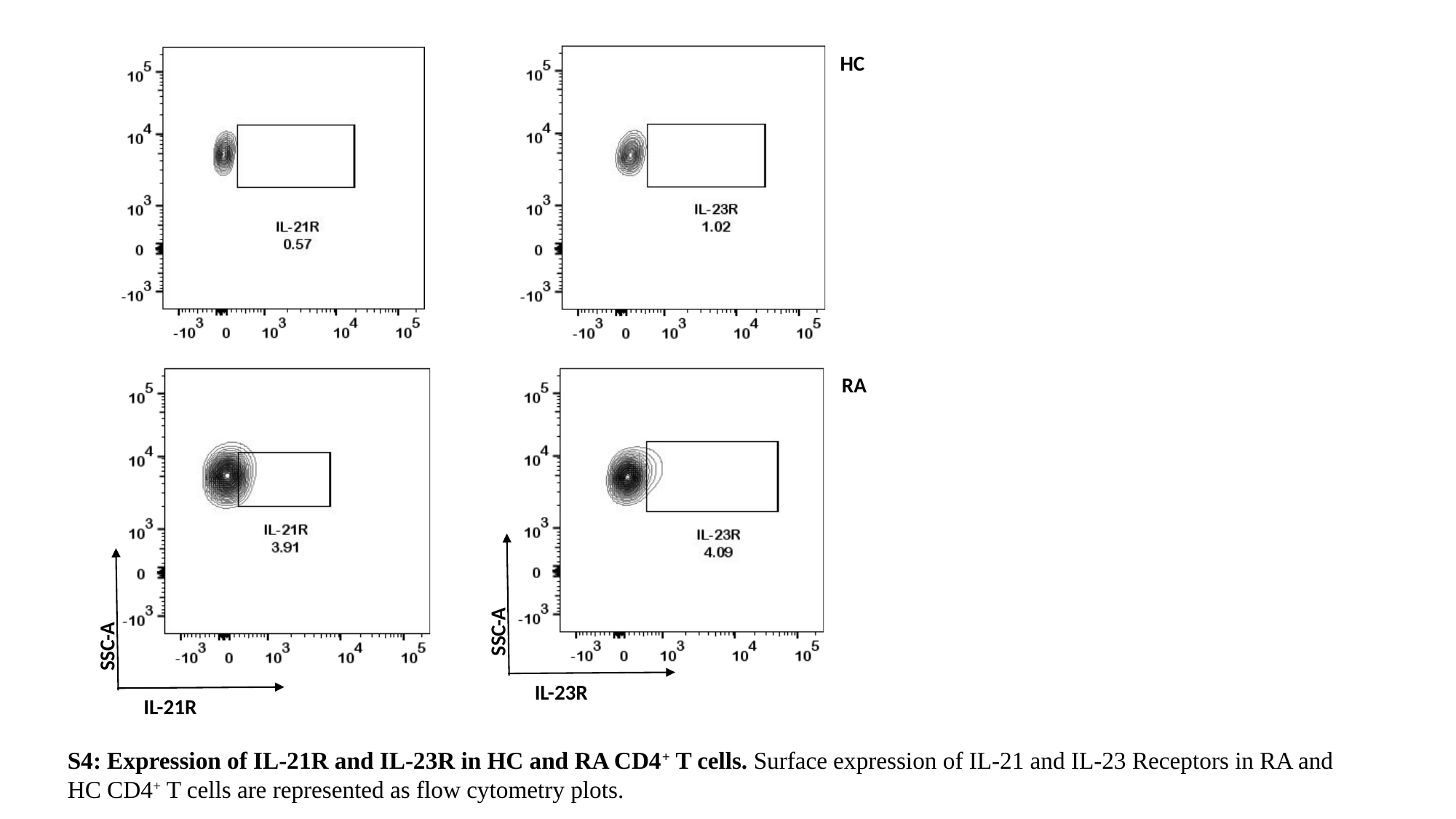

SSC-A
IL-23R
SSC-A
IL-21R
HC
RA
S4: Expression of IL-21R and IL-23R in HC and RA CD4+ T cells. Surface expression of IL-21 and IL-23 Receptors in RA and HC CD4+ T cells are represented as flow cytometry plots.

## Slide 5
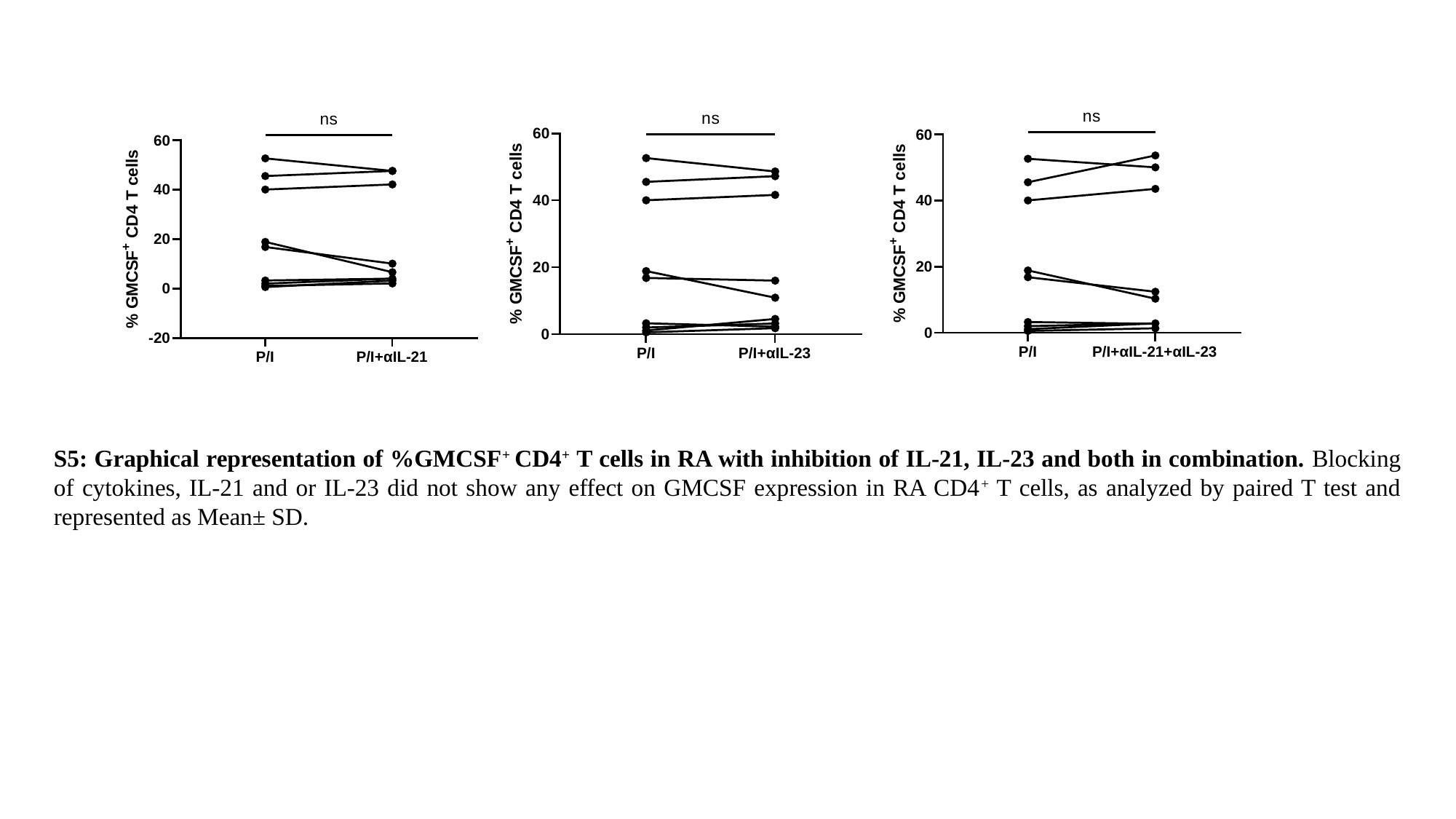

S5: Graphical representation of %GMCSF+ CD4+ T cells in RA with inhibition of IL-21, IL-23 and both in combination. Blocking of cytokines, IL-21 and or IL-23 did not show any effect on GMCSF expression in RA CD4+ T cells, as analyzed by paired T test and represented as Mean± SD.

## Slide 6
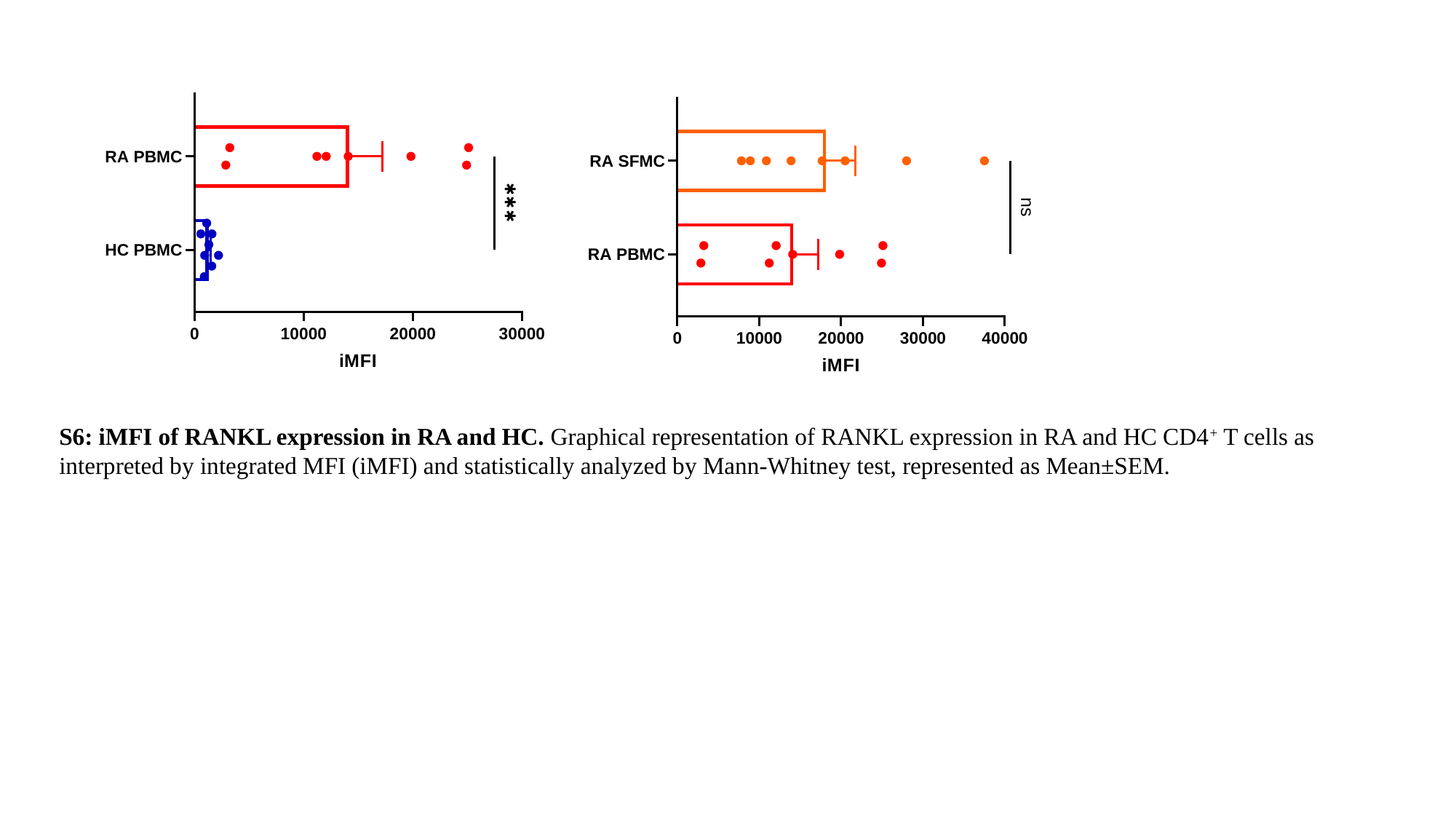

S6: iMFI of RANKL expression in RA and HC. Graphical representation of RANKL expression in RA and HC CD4+ T cells as interpreted by integrated MFI (iMFI) and statistically analyzed by Mann-Whitney test, represented as Mean±SEM.

## Slide 7
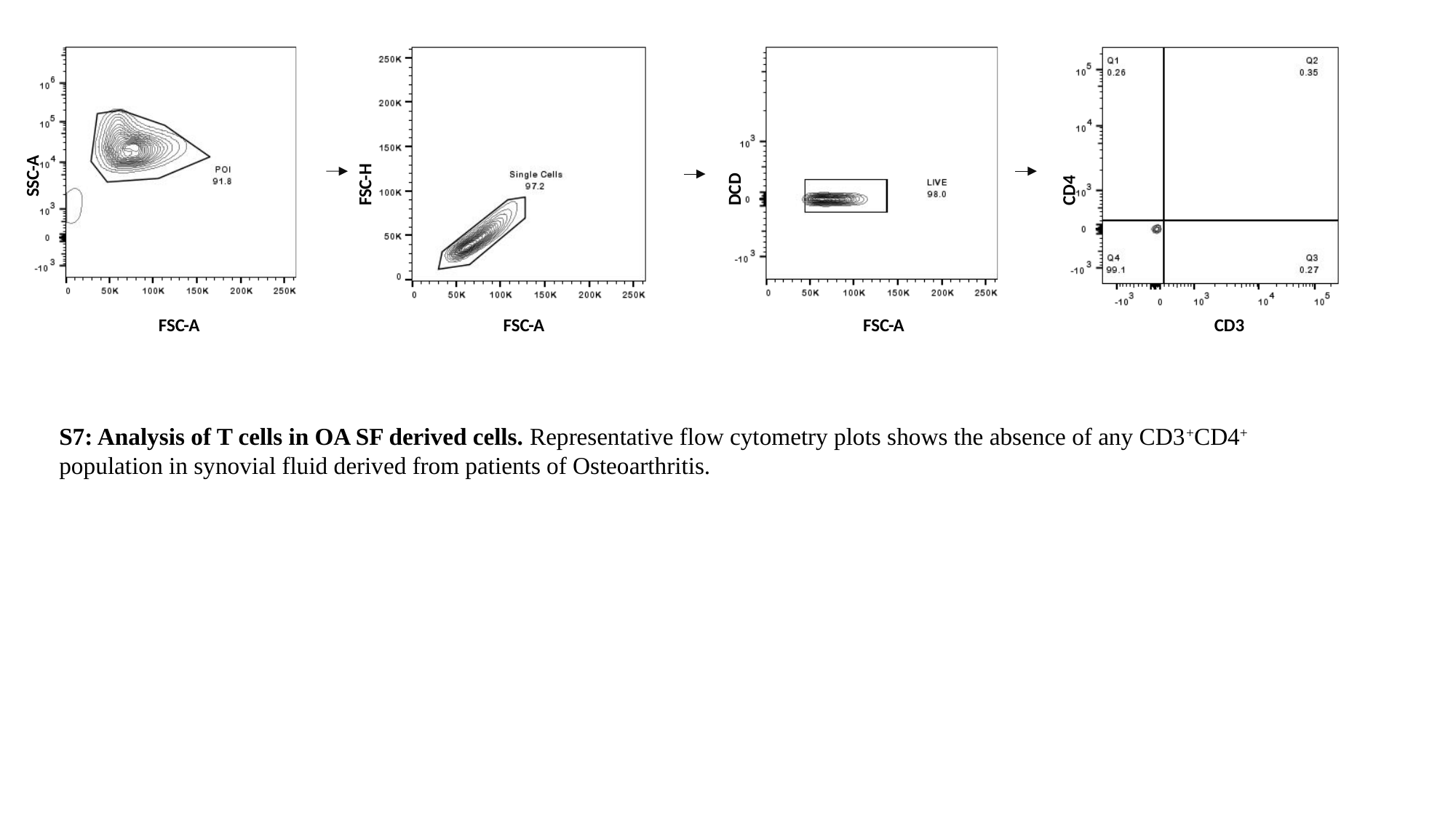

CD4
DCD
FSC-H
FSC-A
CD3
FSC-A
FSC-A
SSC-A
S7: Analysis of T cells in OA SF derived cells. Representative flow cytometry plots shows the absence of any CD3+CD4+ population in synovial fluid derived from patients of Osteoarthritis.

## Slide 8
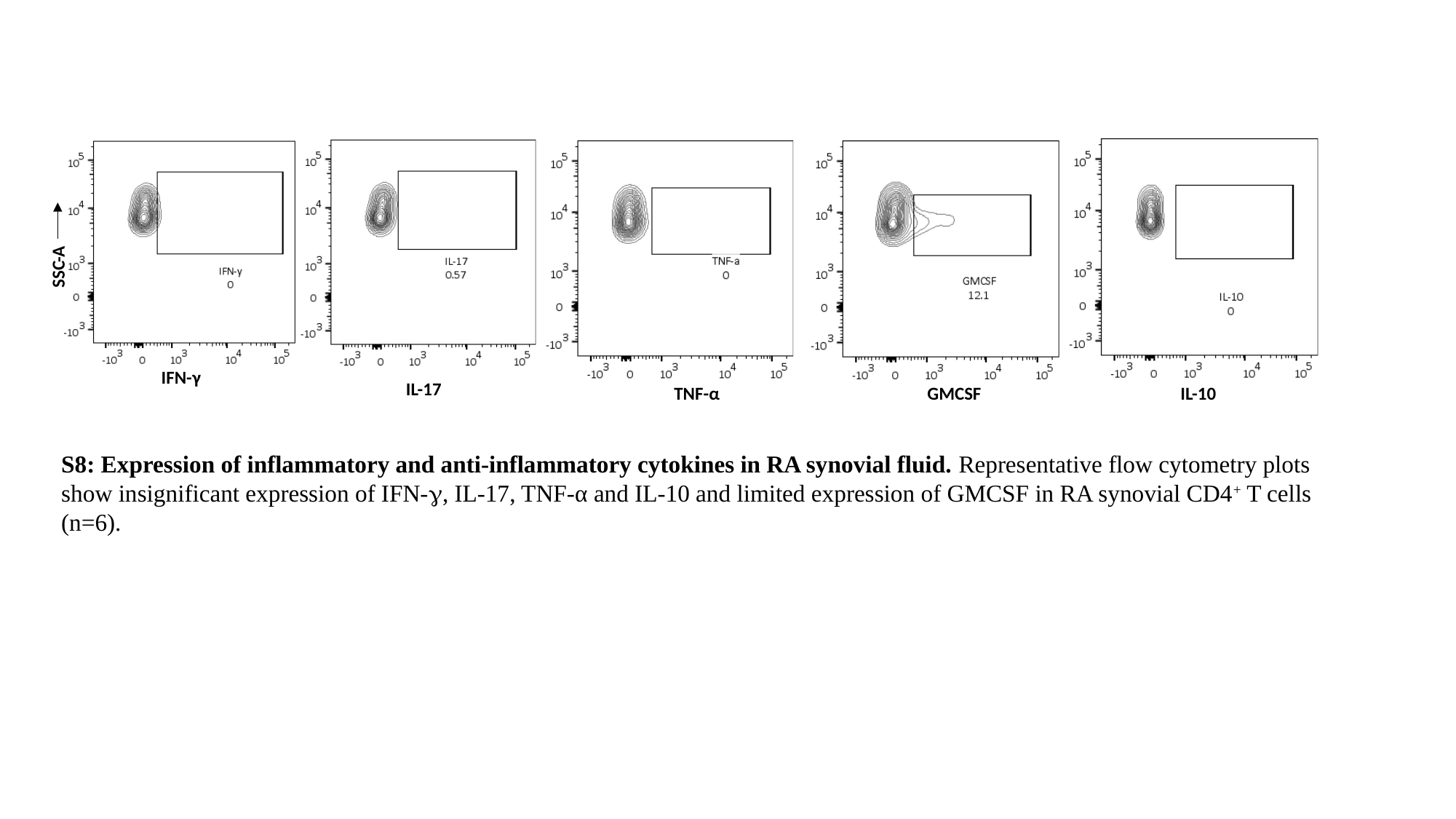

IFN-γ
IL-17
TNF-α
GMCSF
IL-10
SSC-A
S8: Expression of inflammatory and anti-inflammatory cytokines in RA synovial fluid. Representative flow cytometry plots show insignificant expression of IFN-γ, IL-17, TNF-α and IL-10 and limited expression of GMCSF in RA synovial CD4+ T cells (n=6).

## Slide 9
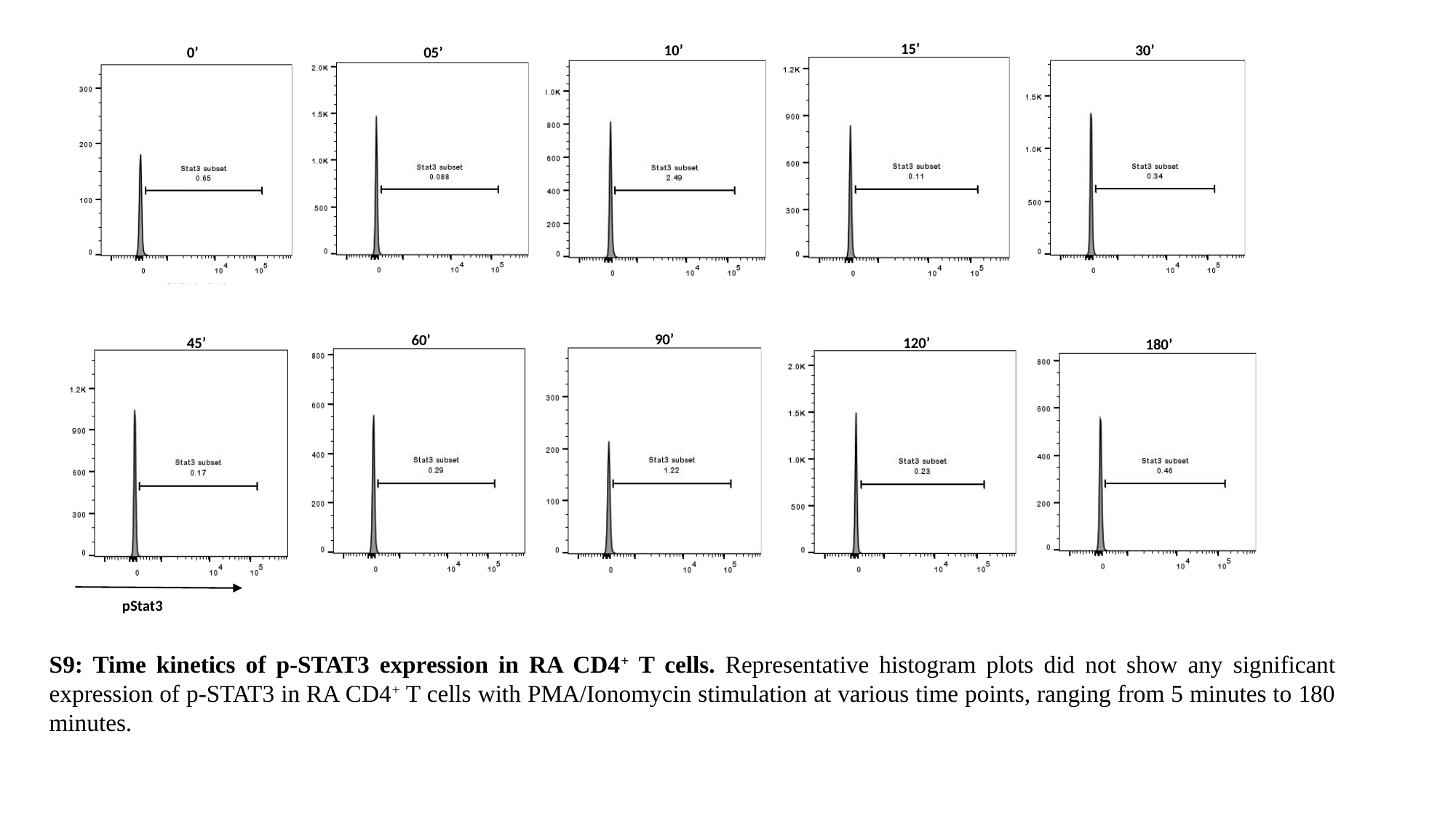

15’
10’
30’
0’
05’
90’
60’
120’
45’
180’
pStat3
S9: Time kinetics of p-STAT3 expression in RA CD4+ T cells. Representative histogram plots did not show any significant expression of p-STAT3 in RA CD4+ T cells with PMA/Ionomycin stimulation at various time points, ranging from 5 minutes to 180 minutes.

## Slide 10
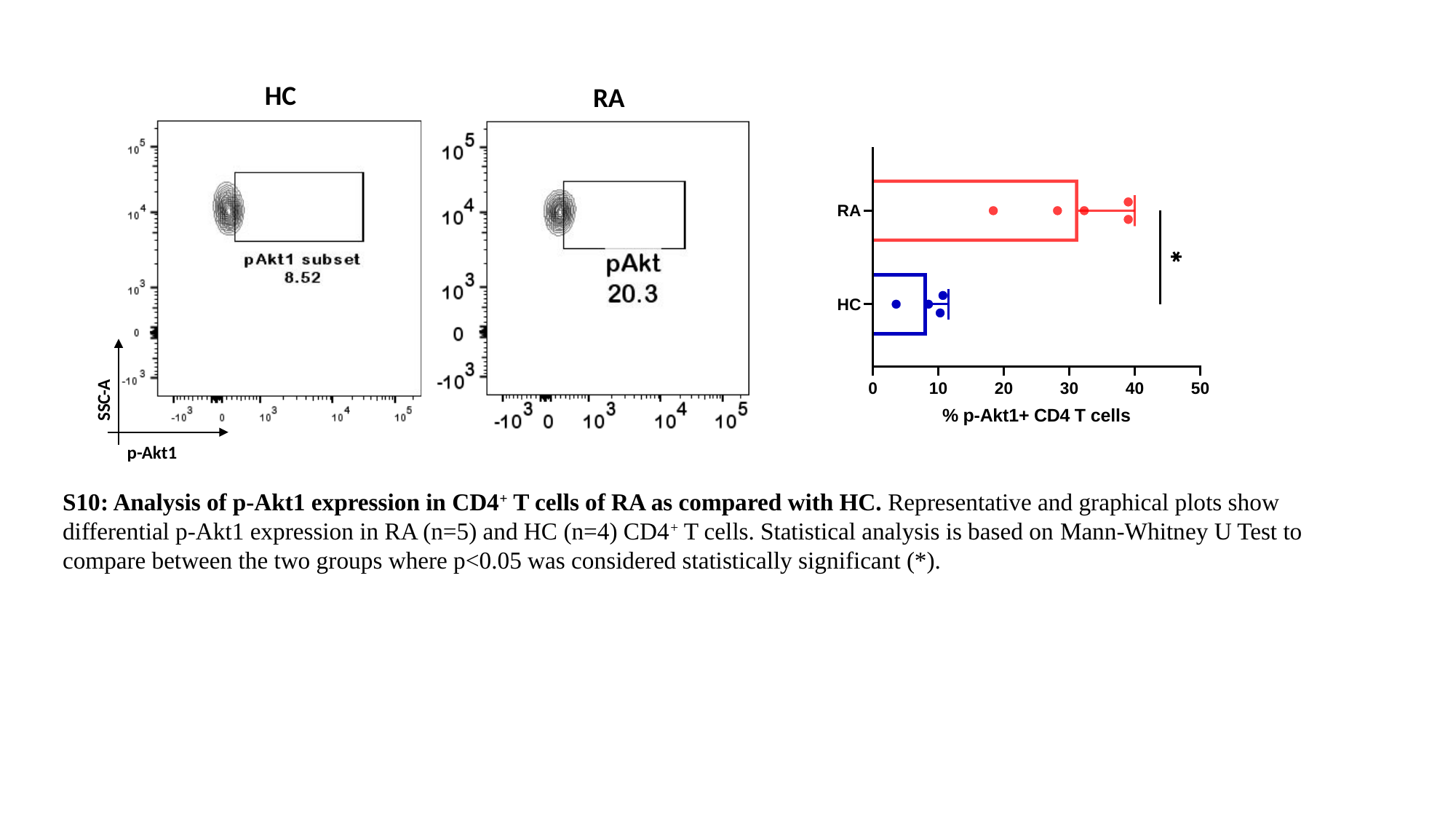

HC
RA
SSC-A
p-Akt1
S10: Analysis of p-Akt1 expression in CD4+ T cells of RA as compared with HC. Representative and graphical plots show differential p-Akt1 expression in RA (n=5) and HC (n=4) CD4+ T cells. Statistical analysis is based on Mann-Whitney U Test to compare between the two groups where p<0.05 was considered statistically significant (*).

## Slide 11
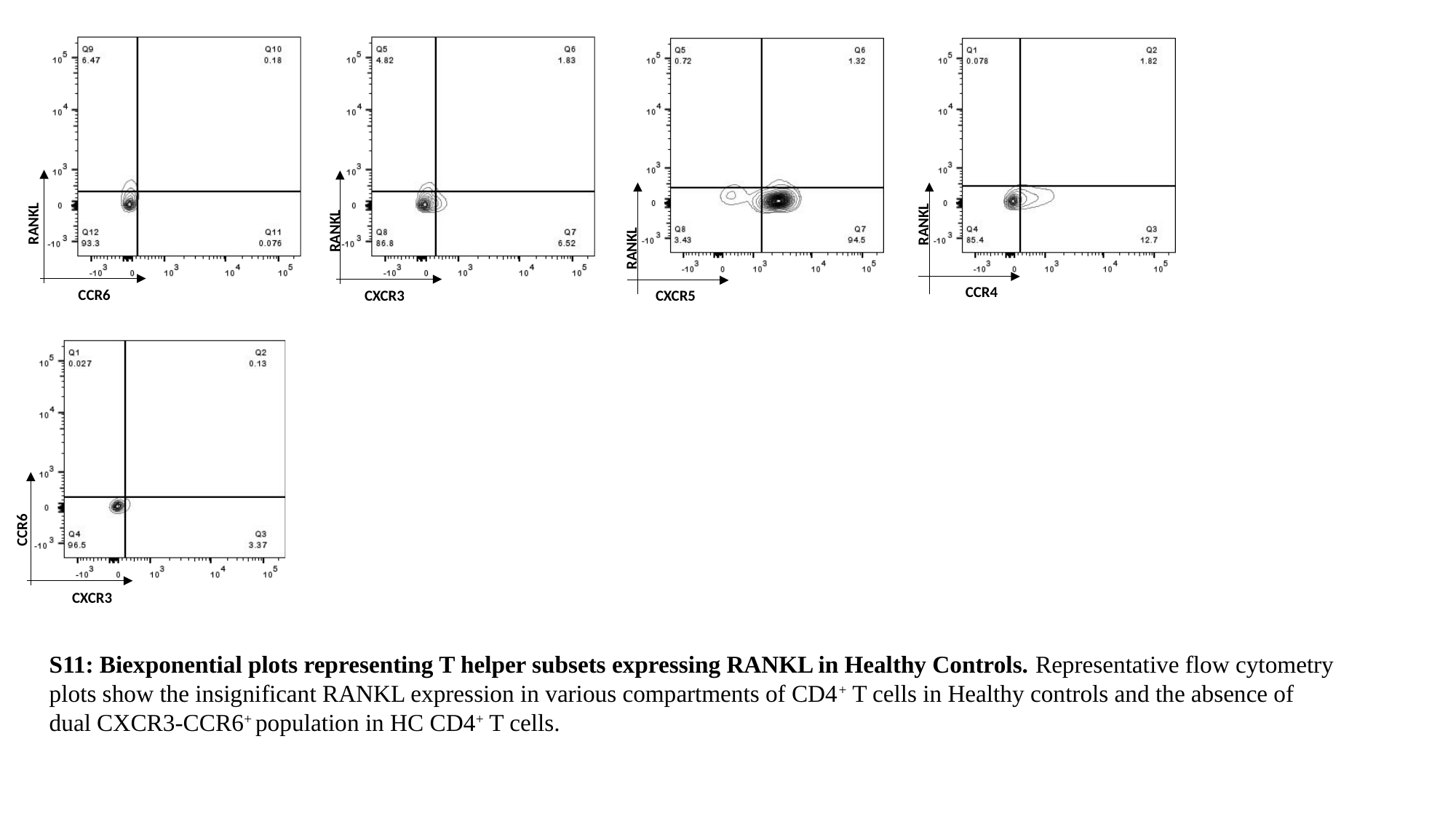

RANKL
RANKL
CCR4
CXCR5
RANKL
CCR6
CXCR3
RANKL
CCR6
CXCR3
S11: Biexponential plots representing T helper subsets expressing RANKL in Healthy Controls. Representative flow cytometry plots show the insignificant RANKL expression in various compartments of CD4+ T cells in Healthy controls and the absence of dual CXCR3-CCR6+ population in HC CD4+ T cells.
