# Supplementary material for: IL-21/23 axis modulates inflammatory cytokines and RANKL expression in RA CD4+ T cells via p-Akt1 signaling
Source: Front Immunol. 2023 Sep 21;14:1235514. doi: 10.3389/fimmu.2023.1235514 (PMC10551441; doi:10.3389/fimmu.2023.1235514)
Supplement: Supplementary file 3 [file Presentation_2.pptx]

## Slide 1
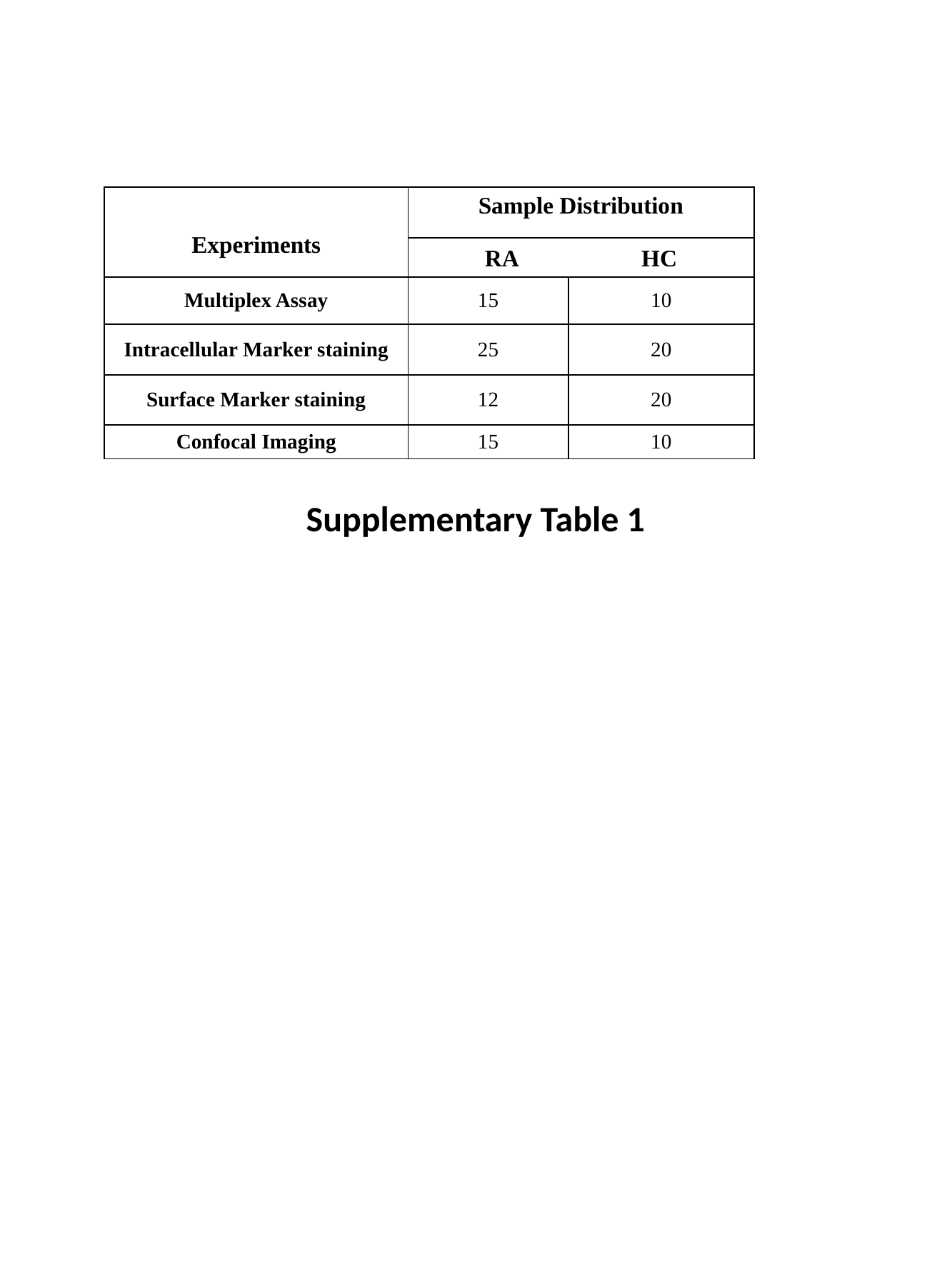

| Experiments | Sample Distribution RA HC | Sample Distribution HC |
| --- | --- | --- |
| Multiplex Assay | 15 | 10 |
| Intracellular Marker staining | 25 | 20 |
| Surface Marker staining | 12 | 20 |
| Confocal Imaging | 15 | 10 |
Supplementary Table 1
